# The Dimerization State of the Mammalian High Mobility Group Protein AT-Hook 2 (HMGA2)

**DOI:** 10.1371/journal.pone.0130478

**Published:** 2015-06-26

**Authors:** Lorraine Frost, Maria A. M. Baez, Christopher Harrilal, Alyssa Garabedian, Francisco Fernandez-Lima, Fenfei Leng

**Affiliations:** 1 Biomolecular Sciences Institute, Florida International University, Miami, Florida, United States of America; 2 Department of Chemistry and Biochemistry, Florida International University, Miami, Florida, United States of America; University of South Florida College of Medicine, UNITED STATES

## Abstract

The mammalian high mobility group protein AT-hook 2 (HMGA2) is a chromosomal architectural transcription factor involved in cell transformation and oncogenesis. It consists of three positively charged “AT-hooks” and a negatively charged C-terminus. Sequence analyses, circular dichroism experiments, and gel-filtration studies showed that HMGA2, in the native state, does not have a defined secondary or tertiary structure. Surprisingly, using combined approaches of 1-Ethyl-3-(3-dimethylaminopropyl)carbodiimide hydrochloride (EDC) chemical cross-linking, analytical ultracentrifugation, fluorescence resonance energy transfer (FRET), and mass spectrometry, we discovered that HMGA2 is capable of self-associating into homodimers in aqueous buffer solution. Our results showed that electrostatic interactions between the positively charged “AT-hooks” and the negatively charged C-terminus greatly contribute to the homodimer formation.

## Introduction

The mammalian high mobility group protein AT-hook 2 (HMGA2) is a nonhistone chromosomal protein expressed almost exclusively in undifferentiated mesenchymal cells [1]. Disruption of the normal expression pattern of HMGA2 causes deregulation of the cell growth and development [1,2]. Work from Chada’s laboratory showed that *Hmga2* knock-out mice developed pygmy phenotype [1]. These mutant mice were severely deficient in fat cells and other mesenchymal tissues. The same group also demonstrated that disruption of *Hmga2* gene caused a dramatic reduction in obesity of leptin-deficient mice (*Lep* ^*ob*^ */Lep* ^*ob*^) in a gene dosage dependent manner: *Hmga2* ^*+/+*^ *Lep* ^*ob*^ */Lep* ^*ob*^ mice weighed over three times more than *Hmga2* ^*-/-*^ *Lep* ^*ob*^ */Lep* ^*ob*^ animals, and the weight of *Hmga2* ^*+/-*^ *Lep* ^*ob*^ */Lep* ^*ob*^ mice was in between [3]. These results suggest that HMGA2 plays an important role in fat cell proliferation and may be a target for the treatment of obesity [3,4]. HMGA2 was also directly linked to oncogenesis [5–7]. Aberrant expression of the protein has been attributed to the formation of various tumors. These tumors include benign tumors, such as lipomas [8], uterine leiomyomas [9], and fibroadenomas [10], and malignant tumors, such as lung cancer [11–13], breast cancer [14], prostate cancer [15], and leukemia [16]. Interestingly, the expression level of HMGA2 often correlates with the degree of malignancy, the existence of metastasis, and a poor prognosis [17,18]. These results suggest that HMGA2 is a potential therapeutic target of anti-cancer drugs [19,20]. Additionally, more recent studies showed that HMGA2 was involved in human height [21,22], stem cell youth [23], and human intelligence [24]. The multi-functionalities of HMGA2 make it one of the most intriguing proteins studied so far.

HMGA2 is an architectural transcription factor [25] that binds to and modulates DNA conformation, thus providing a framework for subsequent transcriptional activities. It binds to AT-rich DNA sequences using three “AT-hook” DNA binding domains that contain a unique palindrome sequence, PRGRP, flanked on each side by one or two positively charged amino acid residues [26,27]. The DNA binding domain, in the absence of DNA, is unstructured [28,29]. However, when it binds to the minor groove of AT-rich DNA sequences, it adopted a defined conformation [27,28]. This disordered-to-ordered structural transition provides a unique opportunity for it adapting to different AT-rich DNA context and participating in various nuclear activities [6]. More recently, utilizing a PCR-based systematic evolution of ligands by exponential enrichment (SELEX) procedure, we identified two consensus sequences for HMGA2: 5′-ATATTCGCGAWWATT-3′ or 5′-ATATTGCGCAWWATT-3′, where W is A or T [30]. Our results showed that the three segments in the consensus sequences (two AT-rich segments and one GC-rich segment) are required for high-affinity binding; mutations of these sequences significantly reduced the DNA binding affinity of HMGA2. These results indicate that HMGA2 does not randomly recognize any AT-rich sequences. In contrast, it binds and bends specific AT-rich DNA sequences [30,31].

One intriguing feature of HMGA2 is the asymmetric charge distribution over its primary structure. In solution, HMGA2 may self-associate to homodimers or homooligomers through electrostatic interactions, and may bind to DNA as a homodimer. In this paper we employed a combination of biophysical and biochemical approaches demonstrating that HMGA2, an intrinsically disordered/unstructured protein (IDP), is capable of self-associating into homodimers in aqueous buffer solution.

## Materials and Methods

### Proteins and reagents

HMGA2 was purified as described previously [26]. The mutant proteins HMGA2Δ95–108 and C41G were made by a PCR-based site directed mutagenesis and purified as described previously [26]. A peptide containing the negatively charged C-terminus (H-CETEETSSQESAEED-OH)), therefore named the C-terminal peptide (CTP), was custom-synthesized by Advanced ChemTech, Inc. Tetramethylrhodamine-5-maleimide (TMR), fluorescein-5-maleimide (FM), and tris-(2-carboxyethyl)phosphine hydrochloride (TCEP) were from Molecular Probes, Inc. Sephacryl S-100 HR and low molecular weight gel filtration calibration kit were from Amersham Biosciences. 1-ethyl-3-[3-dimethylaminopropyl]carbodiimide hydrochloride (EDC) was from Pierce Biotechnology, Inc.

### Circular dichroism (CD) spectra

Solutions containing 10 μM of HMGA2 in BPES buffer (6 mM Na_2_HPO_4_, 2 mM NaH_2_PO_4_, 1 mM Na_2_EDTA, and 185 mM NaCl, pH 7.0) were used for CD measurement. CD spectra were recorded at 24°C on a Jasco J-720 spectropolarimeter. The molar ellipticity ([θ]) was calculated from the equation [θ] = 100θ/cl, where θ is the measured ellipticity in degree, c is the concentration, and l is the path length. Δε was calculated from the equation [θ] = 3298Δε.

### EDC chemical cross-linking experiments

HMGA2 was dialyzed extensively against MES buffer (10 mM MES, pH 5.5, 50 mM NaCl) and then incubated with 2 mM or the indicated amount of freshly made EDC for 2 hours at 24°C. The reaction was stopped by adding β-mercaptoethanol (20 mM, final concentration) or Tris base (100 mM, final concentration). The protein samples were purified using a pre-equilibrated Sephadex G-50 gel filtration column and analyzed by electrophoresis in 15% SDS-PAGE gels.

### Gel filtration experiments

Proteins were resolved in a Sephacryl S-100 HR filtration column (1×50 cm) equilibrated with BPES buffer. The experiments were performed at 24°C using gravitational force to elute the proteins. A low molecular weight gel filtration calibration kit containing five protein standards, ribonuclease A (M_W,_ 13,700), chymotrypsinogen A (M_W,_ 25,000), ovalbumin (M_W,_ 43,000), albumin (M_W,_ 67,000), and Blue Dextran 2000 was used to calibrate the column in three different runs. The fractions at 0.5 ml each were collected; the elution volumes (V_e_) of the proteins were determined by UV absorbance. The Stokes radius (R_S_) of HMGA2 was estimated from a plot of the V_e_ of the four standard proteins vs. their Stokes radii (Ribonuclease A, 16.4 Å; Chymotrypsinogen A, 20.9 Å; Ovalbumin, 30.5 Å; Albumin, 35.5 Å). The apparent molecular weight of HMGA2 was also estimated by a similar method. To determine whether the CTP binds to HMGA2Δ95–108, the CTP-TMR was incubated with HMGA2Δ95–108 for 30 minutes at 24°C, and loaded and resolved by the Sephacryl S-100 HR filtration column (1×50 cm) equilibrated with BPES buffer.

### Sedimentation velocity analysis

HMGA2 was equilibrated by dialysis with BPES buffer. Sedimentation velocity experiments were conducted at 20°C in Beckman Instruments XLI located in the National Analytical Ultracentrifugation Facility at the University of Connecticut Biotechnology Center. The program Sednterp [32] was used to calculate the following physical constants: M_seq_ = 11,819 Dalton, ${\bar{\nu }}_{20^{0}}=0.7123$ g/ml, $\rho_{20^{0}}=1.00712$ g/ml, and $\eta_{20^{0}}=0.01002$ poise, where M_seq_ is the molecular weight calculated from the sequence; ${\bar{\nu }}_{20^{0}}$, $\rho_{20^{0}}$, $\eta_{20^{0}}$ are the partial specific volume, the buffer density, and the buffer viscosity, respectively, at 20°C. A stock solution containing 270 μM of HMGA2 was used to prepare three concentrations of the protein containing, respectively, 135, 45, and 13.5 µM of HMGA2. These dilutions were then used in a sedimentation velocity run, using the 4-hole rotor at 20°C and 55,000 rpm. Synthetic boundary cells (cuvettes in the centerpiece) were loaded with 420 µl of buffer and 410 µl of the appropriate sample solution. The cells were placed in the rotor and accelerated to 12,000 rpm while monitoring the transfer of the excess buffer in each cell. Subsequently, the run was stopped and the rotor was gently inverted to thoroughly mix the contents of the cells. The rotor was then equilibrated in a vacuum at 20°C and after a period of 30 min at 20°C the rotor was accelerated to 55,000 rpm. Interference scans were acquired at 1 min intervals for approximately 6 hours. The data was fitted to two programs Sedfit and Sedanal to determine the protein’s sedimentation coefficient and molecular weight, which can be used to model the protein’s shape using the program Sednterp.

### Labeling of HMGA2 and CTP by TMR or FM

Since HMGA2 and the CTP each contain a cysteine residue, they were labeled using TMR or FM. For labeling, 100 μM of HMGA2 or the CTP was incubated with 200 μM TMR or FM in the presence of 400 μM TCEP in 50 mM phosphate buffer (pH 7.2) plus 20 mM NaCl for 2 hours at 24°C. HMGA2-TMR or HMGA2-FM was purified by two consecutive passages through pre-equilibrated Sephadex G-50 spin columns and dialyzed extensively against the buffer used for labeling. To purify the CTP-TMR, the labeling mixture was loaded onto a pre-equilibrated SP-sepharose column (1 ml) and eluted with 50 mM phosphate buffer (pH 7.2) plus 500 mM NaCl. An extinction coefficient of 95,000 cm^-1^M^-1^ at 541 nm in methanol was used to determine the TMR concentration. An extinction coefficient of 83,000 cm^-1^M^-1^ at 492 nm in Tris-HCl (pH 9.0) was used to determine the FM concentration.

### Steady-State Fluorescence Measurements

Fluorescence spectra of HMGA2-FM were acquired using a Jobin Yvon FluoroMax-3 spectrofluorometer with excitation wavelength of 492 nm. In the fluorescence resonance energy transfer (FRET) titration experiment, 20 nM of HMGA2-FM in Tris buffer (50 mM Tris-HCl (pH 8.0), 50 or 200 mM NaCl) was titrated by increasing concentrations of HMGA2-TMR. The fluorescence spectra were recorded from 500 to 600 nm.

### Trapped Ion Mobility Spectrometry—Mass spectrometry studies (TIMS-MS)

Details regarding the TIMS operation and specifics compared to traditional IMS can be found elsewhere [33–37]. Briefly, in TIMS mobility separation is based on holding the ions stationary using an electric field against a moving gas. The separation in a TIMS device can be described by the center of the mass frame using the same principles as in a conventional IMS drift tube [38]. The TIMS analyzer was coupled to a maXis Impact Q-UHR-ToF (Bruker Daltonics Inc., Billerica, MA). Data acquisition was controlled using in-house software, written in National Instruments Lab VIEW (2012, v. 12.0f3), and synchronized with the maXis Impact acquisition program. TIMS separation was performed using nitrogen as a bath gas at ca. 300 K and typical P_1_ and P_2_ values are 2.6 and 1.0 mbar, respectively. The same RF (880 kHz and 200–350 Vpp) was applied to all electrodes including the entrance funnel, the mobility separating section, and the exit funnel. Protein samples were prepared at 1, 10, 50 and 100 μM concentration using HPLC grade solvents from Thermo Fisher Scientific Inc. (Waltham, MA). An electrospray ionization source (ESI, Bruker Daltonics Inc., MA) was used for all analyses in positive ion mode.

## Results

### HMGA2 is an intrinsically disordered/unstructured protein (IDP)

**Fig 1:**
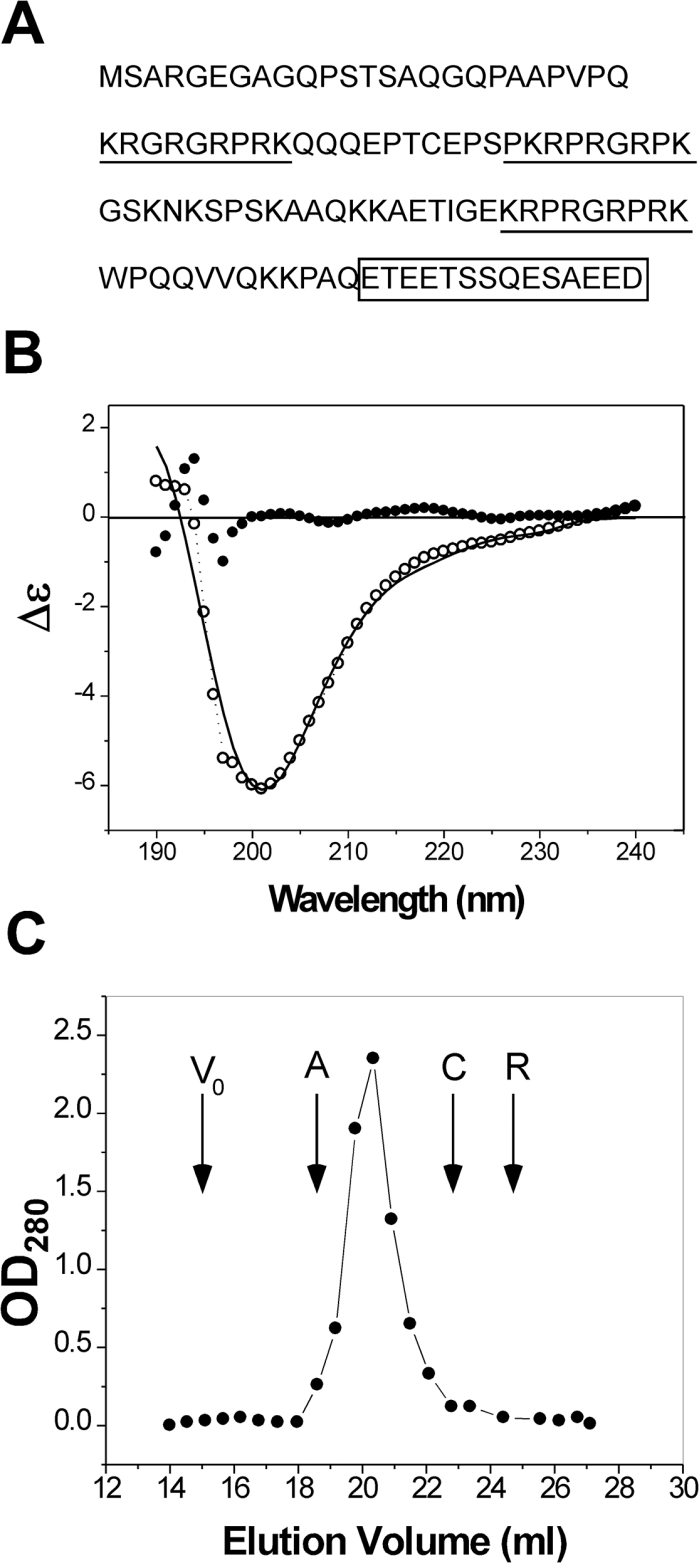
(A) Amino acid sequence of mouse HMGA2. The positively charged “AT hooks” and the negatively charged C-terminus are highlighted by underlines and a box, respectively. **(B) The experimental (open dots with dotted line) and calculated (solid line) CD spectra of HMGA2 in BPES buffer.** The CD spectra were calculated by the program CONTIN as described in the text. The solid dots are residuals. **(C) Gel filtration chromatography profile of HMGA2 in solution.** Recombinant HMGA2 was resolved by gel filtration in the Sephacryl S-100 column as described under “Materials and Methods.” V_0_ is the void volume; A, C, and R represent the elution volume of albumin, chymotrypsinogen A, and ribonuclease A, respectively.

Mouse HMGA2 is a 108 amino acid residue protein. Inspection of its primary structure gives some unique features. It has 25 basic amino acid residues (either Lys or Arg), 12 acidic amino acid residues (either Glu or Asp), 15 Pro, and 10 Gly. The high contents of the basic amino acid residues make HMGA2 a high isoelectric point protein (the isoelectric point was estimated to be about 11; the estimated net charge at pH 7.0 is about +13). The charge distribution of HMGA2 is asymmetrical, with the positively charged residues mainly concentrated in the center of the sequence and the negatively charged residues at the C-terminus (Fig 1A). Several programs, such as PONDR [39], DISOPRED [40], and GlobPlot [41], were used to predict its secondary structure. All predicted that HMGA2 in the native state does not adopt a defined structure. Indeed, our CD studies showed that HMGA2 is an unstructured protein. Fig 1B shows the CD spectrum of HMGA2 in which the strong peak near 200 nm indicates an unordered conformation. The CD results were further analyzed using three CD analysis programs CONTIN, CDSSTR, and SELCON3 that are included in a downloadable software package CDPro [42]. These analyses showed that about 80% of HMGA2 is unstructured (Fig 1B; S1 Table, Supplementary data). Interestingly, all programs suggest that HMGA2 has about 20% β-sheet component. It is possible that part of HMGA2 has an extended structure similar to β strands. A comparison of the experimental and calculated CD spectra is shown in Fig 1B. Similar results were obtained in our NMR analyses and DSC experiments [29].

HMGA2 was also studied by gel-filtration experiments. Fig 1C shows the gel filtration profile of HMGA2. We calculated the Stokes radius (R_S_) of HMGA2 using globular proteins with known hydrodynamic dimensions as standards. The calculated R_S_ of 30.2 Å for HMGA2 is much too large for a protein with a molecular weight of 12,000 Daltons. In comparison, the R_S_ of ribonuclease A, a monomeric globular protein with a similar MW (13,700 Daltons), is 16.4 Å. These results suggest that HMGA2 is either a homodimer (or homo-oligomer) or an extended unfolded non-globular protein or both. The R_S_ calculated from gel filtration studies is almost the same with that calculated from sedimentation velocity analyses (see below for details). Based on the results discussed above, we conclude that HMGA2 is an IDP. This conclusion is consistent with previous studies, which demonstrated that a similar protein, HMGA1a, is an IDP [28,43].

### HMGA2 is a homodimer in aqueous buffer solution

In the process of purifying and characterizing HMGA2, we found that a small amount, but variable of HMGA2 always migrates as a dimeric form on SDS-PAGE gels (data not shown). We also found that HMGA2 readily forms an interstrand disulfide bond through C41 in the absence of a reducing reagent such as β-mercaptomethanol or DTT. Furthermore, HMGA2 aggregates under certain conditions, e.g. at high protein concentrations or in the presence of nucleic acids. As discussed above, one of the unique characteristics of HMGA2 is the asymmetrical charge distribution in the primary structure. We therefore reasoned that the protein might exist as a homodimer or a homo-oligomer in aqueous buffer solution.

**Fig 2:**
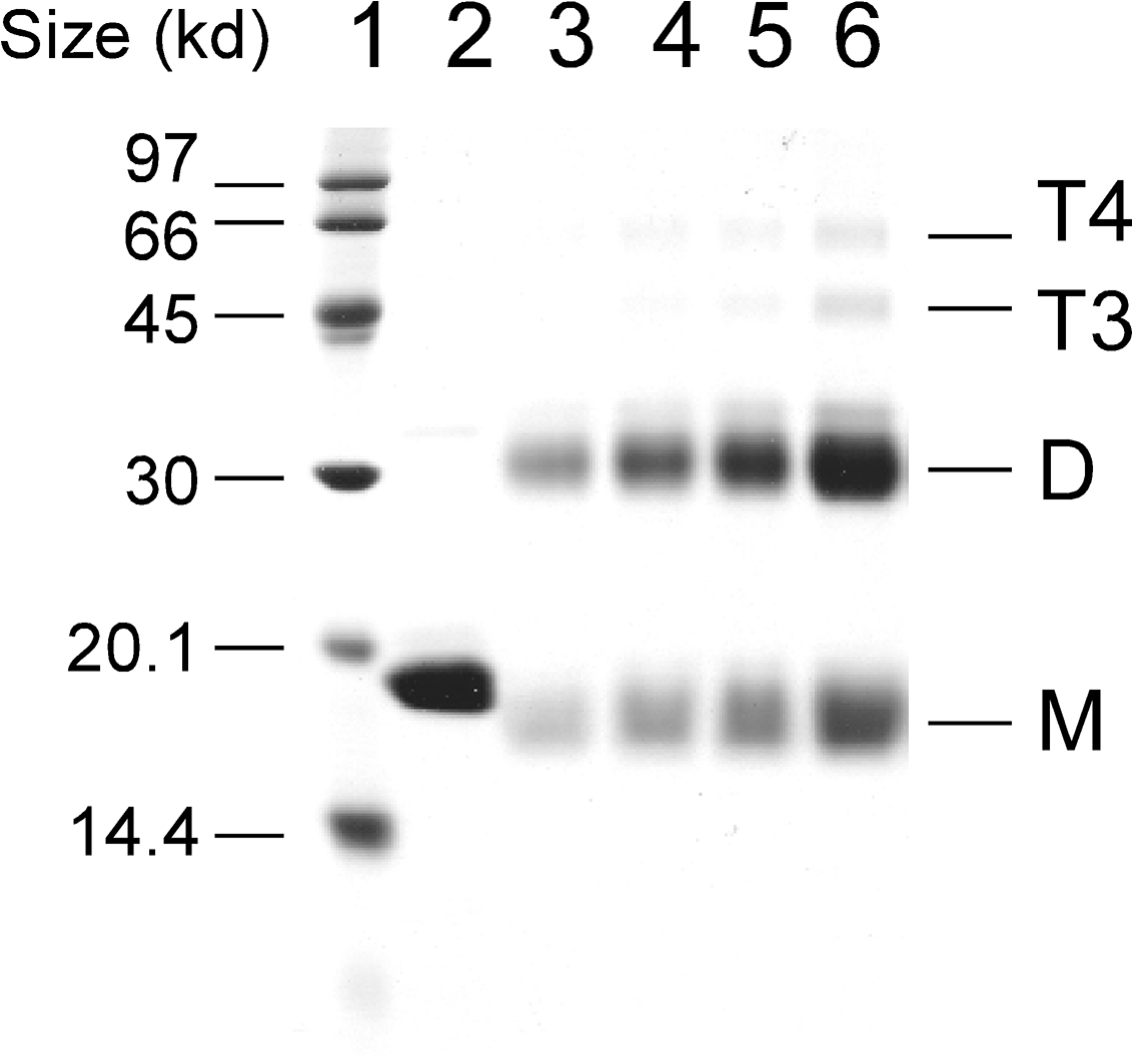
Chemical cross-linking HMGA2 into homodimers with EDC. Chemical cross-linking reactions with EDC in MES buffer were performed as described under “Materials and Methods.” Cross-linked protein samples were analyzed by electrophoresis in a 15% SDS-PAGE gel and stained with Coomassie Brilliant Blue R-250. Lane 1 contained molecular standards; lane 2 contained HMGA2 in the absence of EDC; lanes 3 to 6 contained, respectively, 29, 39, 58, and 116 μM HMGA2 in the presence of 2 mM EDC. M, monomer; D, dimer; T3, trimer; T4, tetramer.

The oligomeric state of HMGA2 was first analyzed using EDC chemical cross-linking experiments. EDC is a zero-length cross-linker that reacts with closely contacted carboxyl and amino groups. For proteins, the carboxyl groups come from the side chains of Glu and Asp residues or from the unmodified C-terminus; the amino groups come from the side chains of Lys residue or the unmodified N-terminus. HMGA2 has 11 Glu and 1 Asp residues of which seven are located in the C-terminus (Fig 1A). It also has 13 Lys residues whose amino groups on the side chain can be cross-linked to the carboxyl groups by EDC. Fig 2 is a typical EDC chemical cross-linking experiment in which EDC efficiently cross-linked HMGA2 into homodimers (compare lanes 3–6 with lane 2, approximately 60–70% of HMGA2 was cross-linked into dimers). These results suggest that some residues between the subunits of HMGA2 homodimers are in very close proximity to one another. Interestingly, after the cross-linking reaction, the protein samples still had a significant amount of monomers that migrated faster than the protein in the control lane (compare lane 2 with lanes 3–6 of Fig 2). These results suggest that the negatively charged C-terminus may also interact with other parts of HMGA2 in the same subunit. When the HMGA2 concentration was increased in the cross-linking reactions, other homo-oligomers were also formed (Lanes 4 to 6 of Fig 2; they were assumed to be trimers or tetramers based on migration rates on the SDS-PAGE gels). Ionic strength has no apparent effects on the EDC chemical cross-linking reactions (data not shown). Similar results were obtained using disuccinimidyl suberate (DSS) and dimethyl suberimidate (DMS) as chemical cross-linking reagents that cross-link two primary amines (data not shown). However, the cross-linking efficiency of DSS and DMS is significantly lower than that of EDC. A cysteineless mutant protein, HMGA2C41G, which cannot form a disulfide bond, was also efficiently cross-linked into homodimers by EDC (data not shown).

HMGA2 as a homodimer was confirmed by sedimentation velocity analyses, which may also be used to derive the self-association constant for the dimerization process. Three concentrations of HMGA2, 13.5, 45, and 135 μM were used. The sedimentation velocity data were first individually fitted using two programs Sedfit V8.7 [44] and Sedanal V3.45 [45] using the model of a single non-interacting discrete species. The results of the individual fits are summarized in Table 1 for the program Sedfit and S2 Table (Supplementary data) for the program Sedanal. The slight trend of increasing sedimentation coefficient with increasing concentration may be indicative of either an association reaction taking place, albeit very weak (this result is consistent with our EDC chemical cross-linking studies in which higher oligomers are formed upon increasing the concentration of HMGA2 (Fig 2)), or possible dissociation of the dimer upon dilution. All attempts to fit the data to either an association or dissociation scheme failed. S1 Fig (Supplementary data) shows the individual fit of the time-difference data for three concentrations of HMGA2 using finite-element numerical solutions of the Lamm equation. The data for all three concentrations was also analyzed globally, i.e. all three data sets fitted simultaneously, using Sedanal V 3.45, which allows the use of fitting models to directly fit the data to various association schemes using multiple data sets. The global fit for the sample datasets to the model of a single ideal species yielded a value for s of 1.711 S [95% confidence intervals: 1.708 S, 1.715 S] and a value for MW of 23.5 kDa. This value for the molecular weight agrees well with that predicted for a homodimer of HMGA2. With the knowledge of *s* and molecular weight of HMGA2, we predicted that HMGA2 is an elongated, non-globular homodimer (Table 2).

**Table 1:** Results for individually fitting the sedimentation velocity data to the model of a single ideal species by the program Sedfit (version 8.7).

Concentration (μM)	*s* (Svedbergs) [95% CI]^a^	Mw (kDa) [95% CI]^b^	RMSD of fit (μM)
13.5	1.641 [1.636, 1.656]	20.6 [19.9, 21.9]	0.15
45	1.682 [1.679, 1.691]	20.5 [20.0, 21.1]	0.26
135	1.705 [1.701, 1.709]	22.1 [21.5, 22.6]	0.82

^a^In this table, *s* represents the sedimentation coefficient of HMGA2 at 20°C; Mw represents molecular weight; RMSD of fit is the root-mean-square deviation of the fit.

^b^Values in parentheses are the 95% confidence interval (CI) for the molecular weight and sedimentation coefficient.

**Table 2:** Hydrodynamic properties of HMGA2 calculated from sedimentation velocity results by program Sednterp.

s_20,W_	1.7731S
^a^Mw	23.5 kDa
Hydration	0.4866 g/g
f/f_0_	1.7987
^a^R_Stokes_	3.39 nm
^b^Mw	45.0 kDa
^b^R_Stoke_	3.02 nm
Cylindrical model, L	18.024 nm
d	1.823 nm
L/d	9.889

^a^The apparent molecular weight (Mw) and Stokes’ radius (R_Stokes_) of HMGA2 was calculated according to sedimentation velocity studies.

^b^The apparent Mw and R_Stokes_ of HMGA2 were calculated according to gel-filtration results. In this table, we assume that HMGA2 is a cylinder. Two parameters, L, the length of the cylinder and d, the diameter, are required to calculate the hydrodynamic model. The following three equations are used to compute L and d: $s=\frac{M(1-\bar{\nu }\rho )}{N_{0}f}$; $f=\frac{3\pi \eta L}{\ln\left\{\frac{L}{d}+\left[0.312+\frac{0.561d}{L}+0.1{\left(\frac{d}{L}\right)}^{2}\right]\right\}}$; $V=\frac{M\delta_{1}}{N_{0}\rho_{}}+\frac{M\bar{\nu }}{N_{0}}=\pi {\left(\frac{d}{2}\right)}^{2}L$, where M, s, f, v-bar, and N_0_ are the molecular weight of the solute (protein), the sedimentation coefficient, the friction coefficient, the partials specific volume of the protein, the buffer density, the buffer viscosity, and Avogadro’s number, respectively.

**Fig 3:**
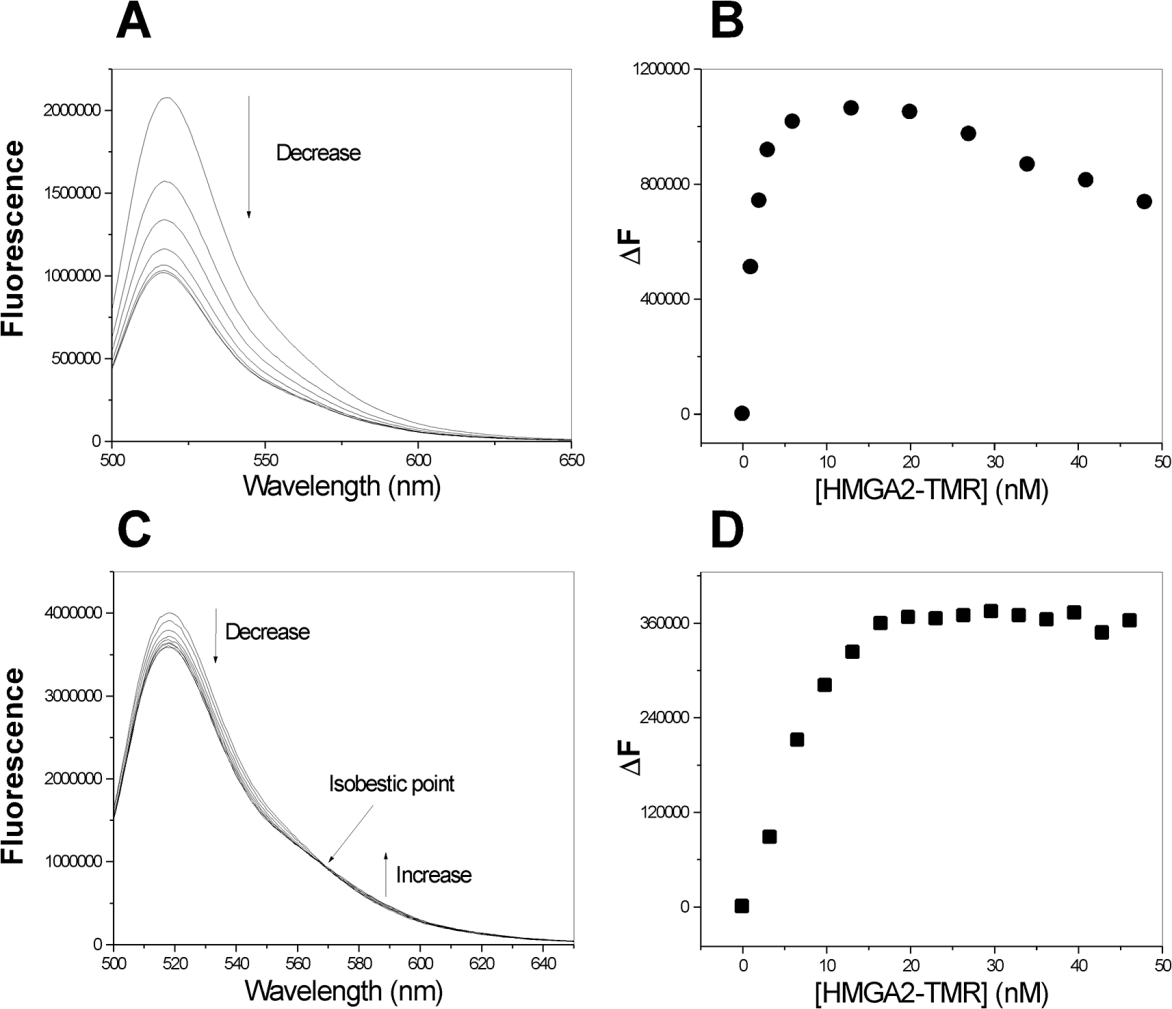
Self-association of HMGA2 was demonstrated by the FRET experiments. **(A)** and **(C)**, respectively, represent fluorescence spectra of HMGA2-FM in the presence of increasing concentrations of HMGA2-TMR in 50 mM Tris-HCl (pH 8.0) and 50 mM **(A)** or 200 mM **(C)** NaCl. **(B)** and **(D)** are the difference in fluorescence intensity at 518 nm (ΔF) as a function of HMG-TMR concentration was shown for the FRET experiment in panel **A** and panel **C** respectively. The fluorescence spectra of HMGA2-FM (20 nM; λ_excitation_ = 492 nm) were recorded as described under “Materials & Methods.”

The self-association of HMGA2 was further studied by fluorescence resonance energy transfer (FRET) titration experiment. In this experiment, HMGA2 was labeled with fluorescein-5-maleimide (FM) or tetramethylrhodamin-5-maleimide (TMR) to produce HMGA2-FM or HMGA2-TMR. If HMGA2 self-associates into homodimers or homooligomers, FRET should be detected using the FM donor/TMR acceptor fluorophore pair. Fig 3 shows the fluorescence spectra of HMGA2-FM in the presence of increasing concentrations of HMGA2-TMR for two different salt concentrations, 50 mM (Fig 3A and 3B) and 200 mM NaCl (Fig 3C and 3D). The fluorescence intensity at 518 nm decreased with increasing HMGA2-TMR concentrations. The decrease saturated at high HMGA2-TMR concentration, indicating that HMGA2-TMR binds to HMGA2-FM. Interestingly, we observed some difference between the two FRET experiments. For the FRET experiment in the presence of 50 mM NaCl, the fluorescence decrease saturated at 5 to 10 nM of HMGA2-TMR and increased again after adding more than 20 nM of HMGA2-TMR (Fig 3B). For the FRET experiment in the presence of 200 mM NaCl, the fluorescence decrease saturated at 20 nM of HMGA2-TMR, indicating a 1:1 molar ratio of HMGA2-FM/HMGA2-TMR (Fig 3B). We also observed the fluorescence increase at 588 nm and an isobestic point for the experiment in the presence of 200 mM NaCl (Fig 3C). In addition, we found that the quench magnitude is different. For the 50 mM case, more than 50% of HMGA2-FM fluorescence was quenched by HMGA2-TMR (Fig 3A); for 200 mM case, only about 10% of HMGA2-FM fluorescence was quenched by HMGA2-TMR (Fig 3C). These results suggest that the self-association of HMGA2 is salt-dependent and the electrostatic interaction plays an important role in the self-association process.

### Mass spectrometry studies

**Fig 4:**
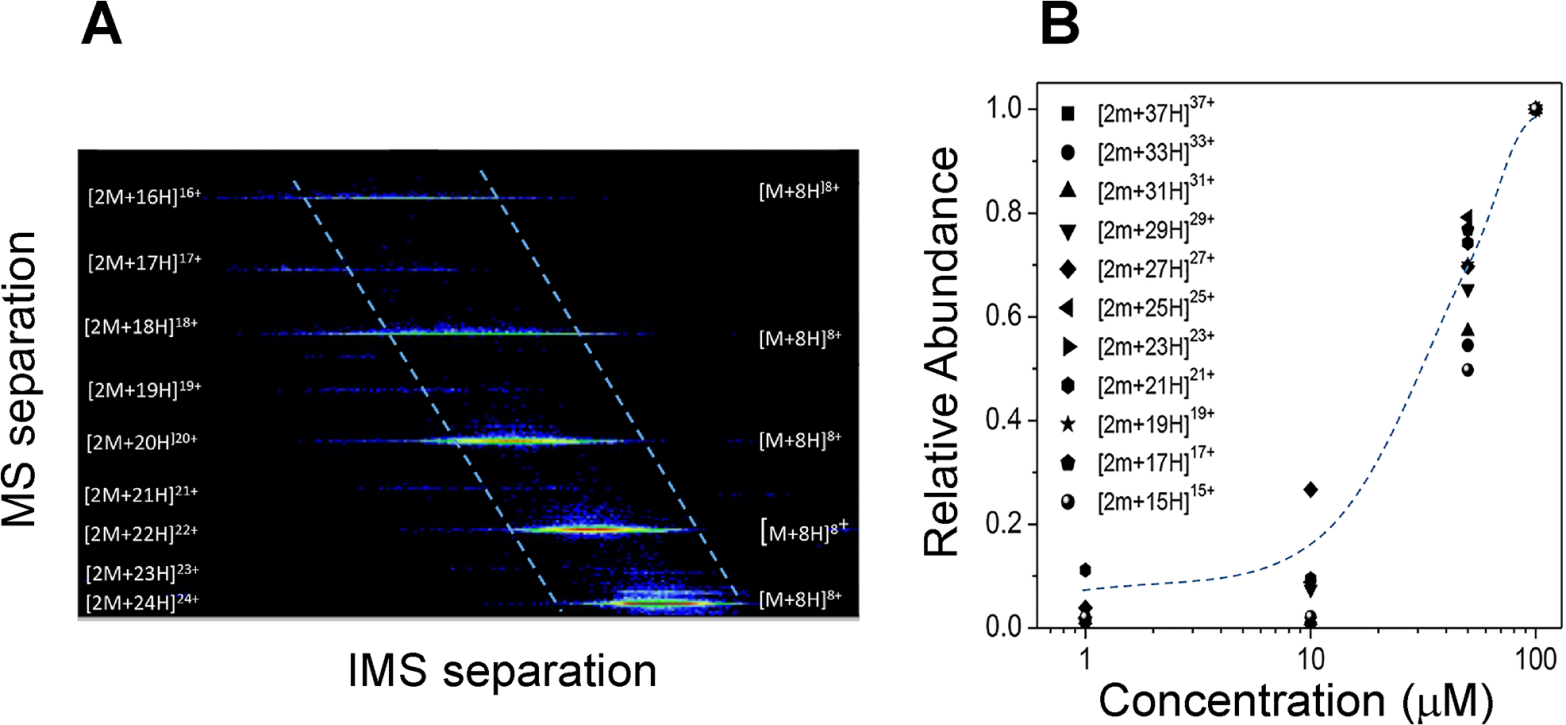
(A) A typical 2D IMS-MS contour plot showing the monomer and dimer signals at 100 μM. Notice the overlap at even charge states in the MS domain for the monomer and dimer peaks. **(B) Relative abundance of the dimer formation as a function of the concentration for the odd charge states.**

The analysis of 2D IMS-MS contour plots (Fig 4) showed the separation in mobility and m/z domains of the [M+nH]^+n^ and [2M+nH]^+n^ monomer and dimer charge state distributions, respectively. Close inspection of the 2D IMS-MS plots shows that the for odd charge state of the [2M+nH]^+n^ series, a clear separation of the dimer component can be achieved, since there is no equivalent m/z on the [M+nH]^+n^ monomer series. In addition, the IMS-MS plots permit the measurements of the relative abundance of each series without the interferences from other m/z. Analysis of the relative abundances of the dimer series relative to the monomer series as a function of the protein concentration in the ESI starting solution shows that as the concentration increases, the dimer formation increases. In particular, dimer observation is significant at concentrations larger than 10 μM. While the mechanism of dimer formation maybe be controversial (i.e., pre-formed in solution or formed during ESI process in the gas-phase), our experimental data from previous experiments (see above) suggest that dimers are pre-formed in solution and remain intact during the ESI process (soft ionization). The lack of trimer, tetramer and higher order oligomers typically observed during ESI condensation at high sample concentration in the IMS-MS data also supports this hypothesis.

### The role of the C-terminus in dimer formation

**Fig 5:**
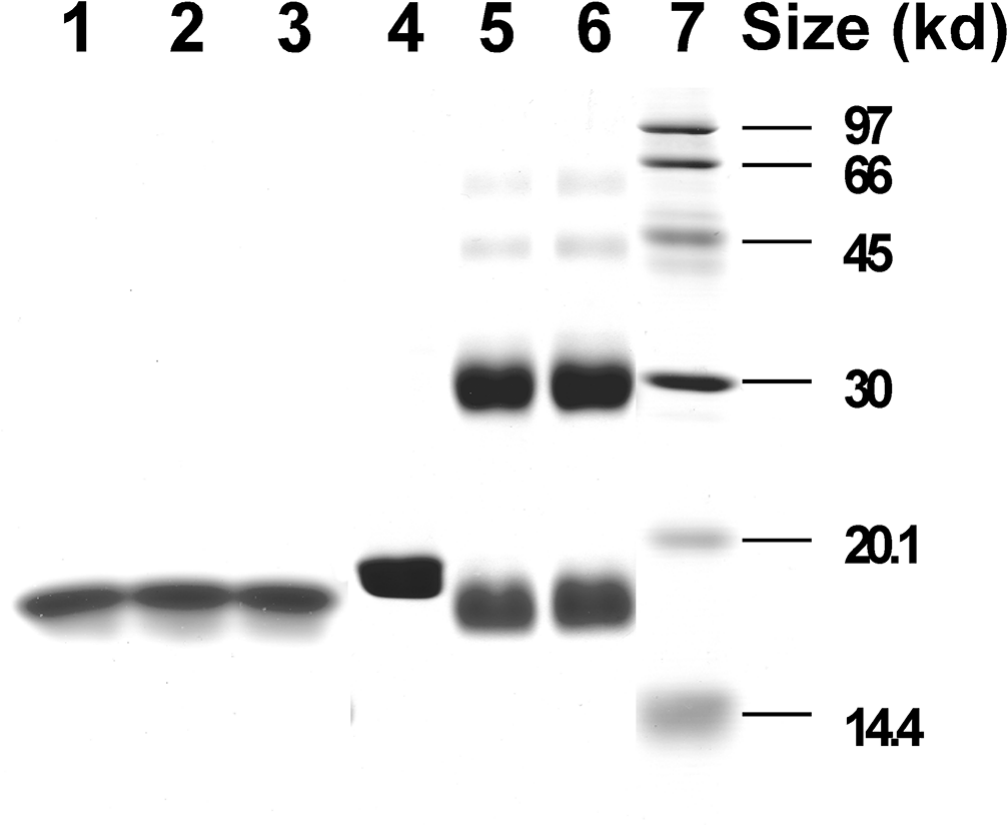
Chemical cross-linking of HMGA2 and HMGA2Δ95–108 with EDC. Chemical cross-linking reactions with EDC in MES buffer were performed as described under “Materials and Methods.” Cross-linked protein samples were analyzed by electrophoresis in a 15% SDS-PAGE gel and stained with Coomassie Brilliant Blue R-250. Lane 1 contained HMGA2Δ95–108 in the absence of EDC; lanes 2 and 3 contained 40 μM HMGA2Δ95–108 in the presence of 2 mM EDC; lane 4 contained HMGA2 in the absence of EDC; lanes 5 and 6 contained 40 μM HMGA2 in the presence of 2 mM EDC; lane 7 contained molecular standards.

**Fig 6:**
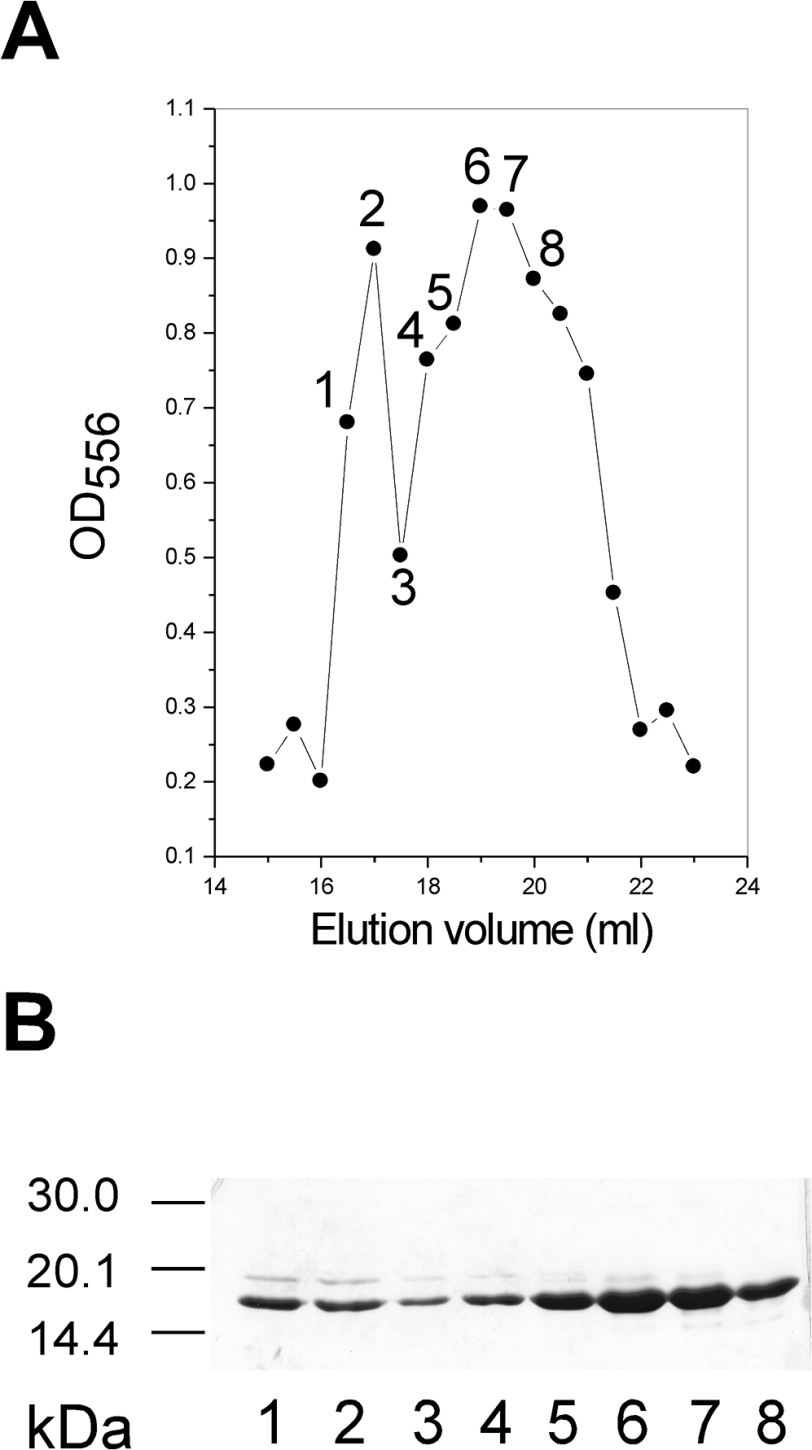
The CTP-TMR and HMGA2Δ95–108 co-eluting in gel-filtration chromatography. The CTP-TMR was prepared as described under “Materials and Methods” and incubated with HMGA2Δ95–108 at 24°C for 30 min in BPES buffer. The CTP-TMR and HMGA2Δ95–108 mixture was then subjected to a Sephacryl S-100 HR filtration column (1×50 cm) equilibrated with BPES buffer. Gel filtration profile of the CTP-TMR binding to HMGA2Δ95–108 was monitored by a graph of OD_556_ versus elution volume (A) and a 15% SDS PAGE gel (B). Lanes 1 to 8 of the SDS-PAGE gel (B) correspond to the fractions 1 to 8 labeled in panel a. Free HMGA2Δ95–108 and the CTP-TMR were eluted at 22 and 30 ml respectively in the column.

We next investigated what factors are critical to HMGA2 homodimer formation. One possible factor is electrostatic interactions between the positively charged “AT-hooks” and the negatively charged C-terminus. We therefore made a mutant HMGA2Δ95–108 that lacks the negatively charged C-terminus, and reasoned that it should not form homodimers. Indeed, our EDC chemical cross-linking experiments showed that HMGA2Δ95–108 could not form homodimers (compare lanes 2 & 3 with lanes 5 & 6 of Fig 5). These results suggest that the negatively charged C-terminus is required for the dimer formation. We then, used tetramethylrhodamine-5-maleimide (TMR) to label a 14 amino acid residue C-terminal peptide (the CTP) of HMGA2 to produce the CTP-TMR. The CTP-TMR was incubated with HMGA2Δ95–108 and subjected to a pre-equilibrated gel filtration column. Fig 6 shows the elution profile of the gel filtration experiment. Our results demonstrated that the CTP-TMR was co-eluted with HMGA2Δ95–108. Interestingly, there are two co-elution peaks (Fig 6). Possibly, the first peak represents two CTP-TMR molecules binding to one HMGA2Δ95–108 and the second peak represents one CTP-TMR molecule binding to one HMGA2Δ95–108. An alternative possibility would be that the first peak contains one molecule of the CTP-TMR binding to two molecules of HMGA2Δ95–108 and the second peak corresponds to one molecule of the CTP-TMR binding to one molecule of HMGA2∆95–108. Further studies are required to determine the binding stoichiometry.

## Discussion

HMGA2, a member of the HMGA family [46] is a classic example of IDP [39,47–49]. It has a characteristic of low overall hydrophobicity and high net charge [48]. Hydrodynamic properties obtained from gel-filtration (Fig 1C) and sedimentation velocity studies (Table 1) showed that HMGA2 is an unfolded, extended, and non-globular protein (Table 2). CD and NMR spectroscopic measurements reveal little secondary or tertiary structure [29]. The key discovery of this study is that HMGA2 is a homodimer under physiological conditions. Not only was a significant amount of HMGA2 cross-linked into dimers by a zero length chemical cross-linker, EDC (Fig 2), but also results from several biophysical methods, such as sedimentation velocity, FRET, and mass spectrometry, demonstrated that it is a stable homodimer in aqueous buffer solution (Figs 3 and 4 and Table 1). An intriguing and important question is: how could an intrinsically unstructured HMGA2 gain quaternary structure, i.e. form homodimers?

**Fig 7:**
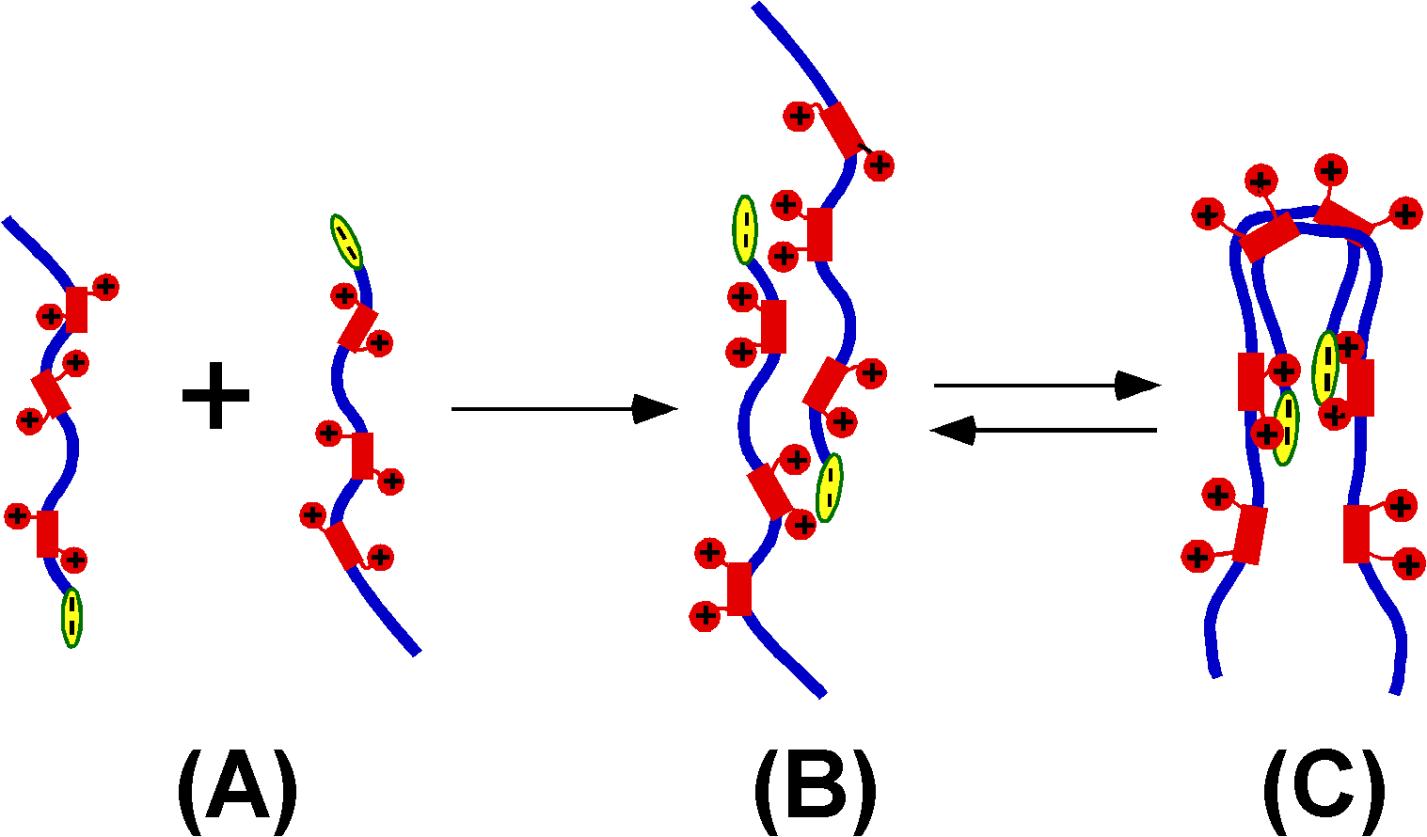
A possible model for the HMGA2 homodimerization. Blue lines represent the protein backbone. Electrostatic interactions between the positively charged “AT hooks” (red rectangle with two red circles) and the negatively charged C-terminus (yellow oval) coordinate the dimer formation. **(A)** represents HMGA2 monomers. **(B)** and **(C)** represent different interchangeable conformations of HMGA2 homodimers. **(C)** is more consistent with our EDC cross-linking and sedimentation velocity results. The HMGA2 homodimers may be an ensemble of different conformers.

Our preliminary results showed that the quaternary structure stems from electrostatic interactions between the positively charged “AT-hooks” and the negatively charged C-terminus. As described above, the charge distribution of HMGA2 is asymmetrical (Fig 1A). This unique property provides an opportunity for the protein to self-associate. As demonstrated in Fig 5, HMGA2Δ94–108, a mutant protein without the negatively charged C-terminus, cannot be cross-linked into dimers by EDC. The CTP containing the negatively charged C-terminus tightly binds to HMGA2Δ94–108 (Fig 6). Moreover, we showed that the negatively charged C-terminus binds to the second “AT-hook” (data not shown) therefore promoting the dimer formation. A possible mechanism would be that the dimer association process is initiated by the charge neutralization and subsequently enforced by the hydrophobic interaction and hydrogen bonding from the peptide backbone. Fig 7 is a schematic graph explaining this mechanism. The “unstructured” monomers (Fig 7A) interact with each other to form a homodimer (Fig 7B and 7C) through electrostatic interactions between the highly charged “AT-hooks” (red rectangle with two red circles) and the highly charged C-terminus (yellow oval). The homodimers do not have regular secondary structure and are composed of an ensemble of different conformers with distinct and dynamic and angles [39,47,50]. They may have local and limited residual structure that is critical for self-association. This dimerization process is reminiscent of the initial step of the amyloid aggregation of a large number of neurodegenerative diseases including Alzheimer’s disease and Parkinson’s disease. In these cases the proteins change their random coil to amyloidenenic β-sheet conformation therefore leading to self-association [51–53]. As demonstrated previously, any generic proteins including intrinsically unstructured proteins have the potential to form amyloid aggregates under suitable conditions [54,55]. This property results from the inherent physicochemical properties of the polypeptide backbone (hydrophobicity and hydrogen bonding) rather than the specific interaction between the side chains [54,56,57]. Although charge was considered to be the key parameter to prevent protein association or aggregation [58], HMGA2 has an asymmetrical charge distribution that would promote self-association. Indeed, recent evidence showed that the electrostatic interaction can promote protein or polypeptide’s self-association. Tjernberg *et al*.[59] showed that peptides as few as 4 residues can form well defined amyloid fibril. Both charge attraction and hydrophobic interaction are required. Goers *el al*. showed that several unstructured polycations, such as spermine, polylysine, polyarginine, and polyethyleneimine tightly bind to -synuclein, an IDP, and catalyze its oligomerization [60]. This process was mediated by the electrostatic interaction between the negatively charged C-terminus and the polycations, and may be enforced by the hydrophobic interaction and hydrogen bonding of the peptide backbone [48]. These studies suggest that the intrinsically “unstructured” proteins or polypeptides can interact with each other to form higher structures.

Our conclusion that HMGA2 is a homodimer in solution is further supported by previous observations. Yie *et al*. showed that HMGA1a, another member of HMGA family, interacts with itself in a glutathione S-transferase (GST)-pull down experiment and the minimal region required for the self-association contains the last basic repeat and the acidic C-terminus (Fig 1 of reference[61]). The self-association may be entirely caused by the electrostatic interaction between the unstructured “AT hooks” and the C-terminus; however, a possible involvement of the well-folded GST in the pull-down experiment would complicate the interpretation of the results. In a separate study, Padmanabhan *et al*. demonstrated that *Myxococcus xanthus* transcription factor CarD, a HMGA-like protein containing both the “AT-hook” DNA-binding domain and the acidic regions, is a homodimer [62]. Sedimentation equilibrium analysis confirmed that CarD is a homodimer. However, because “the inherent difficulty in pinpointing the mechanism of HMGA cooperativity has been noted before and attributed to, among others, its lack of defined structures [62],” it is difficult to determine whether the “AT hooks” and the acidic domain were involved in the dimer formation. In contrast, this study implied that the unstructured “AT-hooks” were not required for the dimer formation. Here, using several biochemical and biophysical methods we demonstrated that the “AT-hook” DNA binding proteins, although without a defined secondary or tertiary structure, form higher structures such as homodimers.

It has been demonstrated recently that other IDPs are also capable of self-associating into homodimers [63–67]. For instance, the cytoplasmic region of the T cell receptor subunit, an IDP is able to self-associate into homodimers [63]. Interestingly, NMR studies showed that the dimerization was not accompanied by a disorder-to-order transition, suggesting that specific interactions exist between two unstructured subunits [68]. Another example is the intrinsically disordered N-terminal domain of ultraspirale from Aedes aegypti (aaUsp-NTD) [67]. In solution, it self-associated into homodimers as well [67]. It is possible that many other IDPs can self-associate into homodimers and the homodimerization is critical for their biological functions.

As mentioned above, HMGA2 is a DNA-binding protein and specifically recognizes the minor groove of AT-rich DNA sequences. Since many promoter regions usually contain multiple AT-rich sites [69,70]. It is possible that HMGA2 cooperatively binds to these regions as a homodimer, and thus modulates the DNA conformation, providing a framework for organizing functional transcription machinery.

## Supporting Information

S1 Fig A plot of the individual fits of the sedimentation velocity data to the model of a single ideal species by the program Sedanal (version 3.45). Panels A to C represent the sedimentation velocity experiments performed for HMGA2 concentrations of 135, 45, and 13.5 μM, respectively. The sedimentation velocity results are shown by the black dotted lines plotted as the concentration difference between pairs of interference scans against radial distance. The red curves are calculated fits. The blue lines are the residuals. Fifty pairs of scans were used in the fitting but only three pairs are shown.
(TIF)

S1 Table Secondary structure fractions of HMGA2 analyzed by three software programs, CONTIN, CDSSTR, and SELCON3. (DOCX)

S2 Table Results for individually fitting the sedimentation velocity data to the model of a single ideal species by the program Sedanal (version 3.45). (DOCX)
