# Supplementary figures and images for: The Dimerization State of the Mammalian High Mobility Group Protein AT-Hook 2 (HMGA2)
Source: PLoS One. 2015 Jun 26;10(6):e0130478. doi: 10.1371/journal.pone.0130478 (PMC4482583; doi:10.1371/journal.pone.0130478)

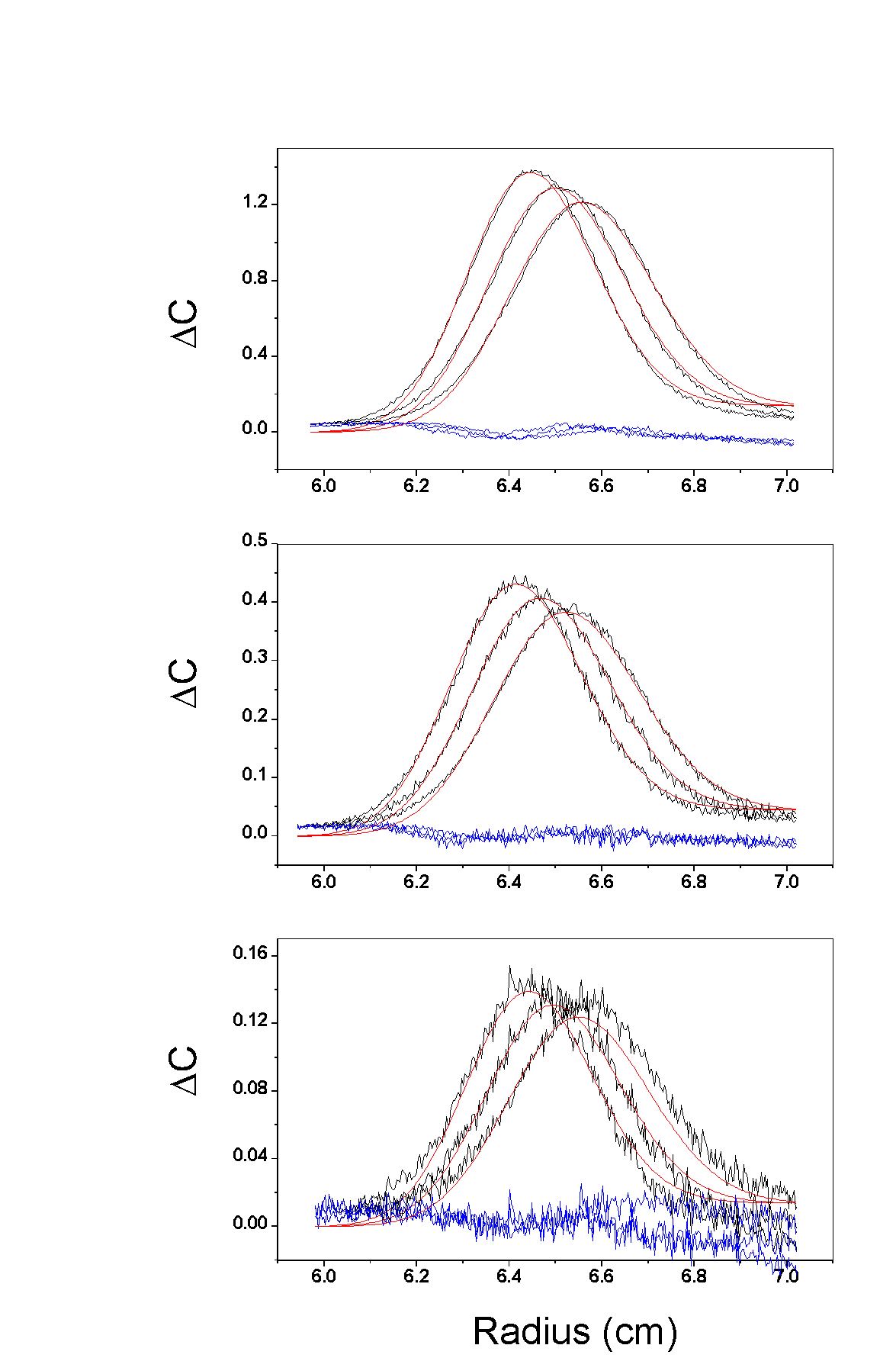

Supplement: S1 Fig — Panels A to C represent the sedimentation velocity experiments performed for HMGA2 concentrations of 135, 45, and 13.5 μM, respectively. The sedimentation velocity results are shown by the black dotted lines plotted as the concentration difference between pairs of interference scans against radial distance. The red curves are calculated fits. The blue lines are the residuals. Fifty pairs of scans were used in the fitting but only three pairs are shown. (TIF) [file pone.0130478.s001.tif]
