# Supplementary material for: The Dimerization State of the Mammalian High Mobility Group Protein AT-Hook 2 (HMGA2)
Source: PLoS One. 2015 Jun 26;10(6):e0130478. doi: 10.1371/journal.pone.0130478 (PMC4482583; doi:10.1371/journal.pone.0130478)
Supplement: S1 Table — (DOCX) [file pone.0130478.s002.docx]

Table S1. Secondary structure fractions of HMGA2 analyzed by three software programs, CONTIN, CDSSTR, and SELCON3.

Methods α_R_ α_D_ β_R_ β_D_ T U δ

CONTIN 0.060 0.148 0.169 0.115 0.215 0.293 0.325

CDSSTR 0.007 0.049 0.226 0.176 0.290 0.240 0.388

SELCON3 0.032 0.093 0.167 0.121 0.230 0.326 2.449

^a^The secondary structures are: α_R_, regular α-helix; α_D_, distorted α-helix; β_R_, regular β-strand; β_D_, distorted β-strand; T, turns; and U, unordered. δ is the RMS deviation calculated by the equation,$\delta=\sqrt{\frac{\Sigma_{i}(f_{i}^{CD}-f_{i}^{X})^{2}}{N}}$, where $f_{i}^{CD}$and $f_{i}^{X}$ are CD and X-ray estimates for secondary structure types of N reference proteins.
