# Supplementary material for: The Dimerization State of the Mammalian High Mobility Group Protein AT-Hook 2 (HMGA2)
Source: PLoS One. 2015 Jun 26;10(6):e0130478. doi: 10.1371/journal.pone.0130478 (PMC4482583; doi:10.1371/journal.pone.0130478)
Supplement: S2 Table — (DOCX) [file pone.0130478.s003.docx]

Table S2. Results for individually fitting the sedimentation velocity data to the model of a single ideal species by the program Sedanal (version 3.45).

Concentration *s* (Svedbergs) Mw (kDa)

(μM) [95% CI]^b^ [95% CI]^b^

13.5 1.665 [1.655, 1.674] 23.5 [22.5, 24.6]

45 1.696 [1.691, 1.700] 22.1 [21.5, 22.5]

135 1.713 [1.709, 1.718] 23.6 [23.2, 24.1]

^a^In this table, *s* represents the sedimentation coefficient of HMGA2 at 20^0^C; Mw represents molecular weight. ^b^Values in parentheses are the 95% confidence interval (CI) for the molecular weight and sedimentation coefficient.
